# Predictors of Colorectal Cancer Screening Rates in Federally Qualified Health Centers: Explicating Organizational Level Factors

**DOI:** 10.1111/1475-6773.70082

**Published:** 2026-01-04

**Authors:** P. J. Zaire, L. H. Smith, J. Hefner

**Affiliations:** ^1^ College of Nursing The Ohio State University Columbus Ohio USA; ^2^ College of Public Health The Ohio State University Columbus Ohio USA

**Keywords:** colorectal cancer screening, federally qualified health centers, historically marginalized, organizational predictors of screening

## Abstract

**Objective:**

To examine changes in colorectal cancer (CRC) screening rates over time and determine organizational‐level factors influencing these shifts.

**Study Settign and Design:**

This longitudinal study used mixed effects models to analyze data from Federally Qualified Health Centers (FQHCs) in the United States (US). Key organizational‐level factors included Patient‐Centered Medical Home (PCMH) recognition and duration, hypertension and diabetes management, and center‐level characteristics such as racial composition, location, and center volume/size.

**Data Sources and Analytic Sample:**

This study used Uniform Data System (UDS) data from 2017 to 2022 for US‐based FQHCs receiving full Public Health Service Section 330 grants and reporting CRC screening measures, excluding school‐based centers, US territories, and look‐alike centers.

**Principal Findings:**

Among the 1282 FQHCs analyzed, CRC screening rates were increasing before the COVID‐19 pandemic but declined during and remain below pre‐pandemic levels. FQHCs with consistent PCMH recognition reported significantly higher screening rates (*β* = 8.50, *p* < 0.001). Screening rates were also positively associated with a higher rate of controlled hypertension (*β* = 0.354, *p* < 0.0001) but lower in FQHCs with larger Black patient populations, Southern locations, and smaller center volume/size.

**Conclusions:**

Consistent PCMH recognition and chronic disease management are essential for improving CRC screening rates in FQHCs. By integrating these population health management strategies, FQHCs can proactively address screening disparities. Prioritizing these organizational‐level approaches may strengthen healthcare equity and expand CRC screening for historically marginalized communities.

**Summary**

- What is known on this topic
  ○ Colorectal cancer (CRC) remains a significant public health concern in the United States, with screening disparities disproportionately affecting underrepresented populations served by Federally Qualified Health Centers (FQHCs).
  ○ CRC screening challenges are multidimensional, impacting every level of the socioecological system.
  ○ Organizational‐level interventions, such as Patient‐Centered Medical Home (PCMH) recognition and chronic disease management, have been associated with improved overall health outcomes.
- What this study adds
  ○ The measurable impact of the duration of PCMH recognition on colorectal CRC screening rates in FQHCs, reinforcing the importance of sustained organizational‐level interventions.
  ○ Highlights the role of chronic disease management, particularly hypertension control, in improving CRC screening rates, emphasizing the need for integrated approaches that address multiple health conditions simultaneously.
  ○ Provides actionable insights for policymakers and healthcare leaders, guiding the development of strategies that strengthen health system accountability, optimize resource allocation, and advance equitable cancer prevention efforts.

## 1 Introduction

Colorectal cancer (CRC) is the second‐leading cause of cancer‐related deaths in the United States (US) when mortality across both men and women is combined [1], and accounted for 11.6% of all cancer treatment costs in 2020 [2], making it a major public health concern. Screening is a key public health tool for CRC prevention and treatment. Early and frequent screening significantly improves outcomes, enabling polyp removal [3] and raising the 5‐year survival rate to 91% [1]. Without timely detection, advanced‐stage diagnoses lead to much lower survival rates. Black Americans, a historically marginalized group, experience disproportionately high CRC mortality rates [4]. This disparity reflects broader systemic barriers that shape CRC screening and treatment access across multiple levels of the socioecological system.

Historically marginalized communities face barriers to CRC screening and treatment across the multi‐level socioecological system, including individual, interpersonal, organizational/community, and societal factors that influence health [5]. Key individual level factors hindering timely screening include an individual's ability to understand and use health information to access screening [6], out‐of‐pocket costs [7], and a lack of knowledge about CRC and the screening process [5, 8]. The interpersonal level reflects the patient‐provider relationship, particularly the quality of communication; receiving a provider recommendation remains one of the most influential predictors of CRC screening [9, 10, 11]. At the level of community and healthcare organizations, screening obstacles include limited clinic capacity, fragmented care, and lack of a usual source of care [12]. Medical mistrust, rooted in historical and ongoing mistreatment of specific communities, also contributes to reduced CRC screening [13]. Societal issues such as health policy gaps, outdated payment models [14], and structural racism [13], further impede CRC screening. Regional disparities in CRC screening, especially in areas where minoritized populations reside, reflect broader societal barriers rooted in structural inequities [13]. While individual and interpersonal barriers to CRC screening have been extensively studied, less is known about how organizational‐level factors, such as organizational resources, chronic disease management, and preventive care models, impact screening rates.

Federally Qualified Health Centers (FQHCs) serve populations disproportionately affected by these challenges, underscoring the urgent need to tackle organizational‐level factors to improve screening rates and advance health equity. FQHCs, with their mission to provide equitable, patient‐centered care, are uniquely positioned to address these systemic challenges. The Patient‐Centered Medical Home (PCMH) model, for example, offers a promising framework for integrating preventive services and chronic disease management [15, 16, 17]. PCMH is a healthcare model designed to improve care coordination, enhance patient outcomes, and streamline healthcare delivery through a team‐based, patient‐focused approach. Emphasizing comprehensive, coordinated, accessible, and continuous care, particularly for managing chronic diseases and preventive services [18]. The impact of long‐term PCMH recognition and other organizational‐level factors on CRC screening remains unclear. The role of chronic disease management, particularly population health management strategies used to coordinate care, monitor outcomes, and engage patients, is similarly underexplored in the context of CRC screening, even though such strategies are routinely employed to support screening delivery and improve overall health outcomes [19].

**FIGURE 1:**
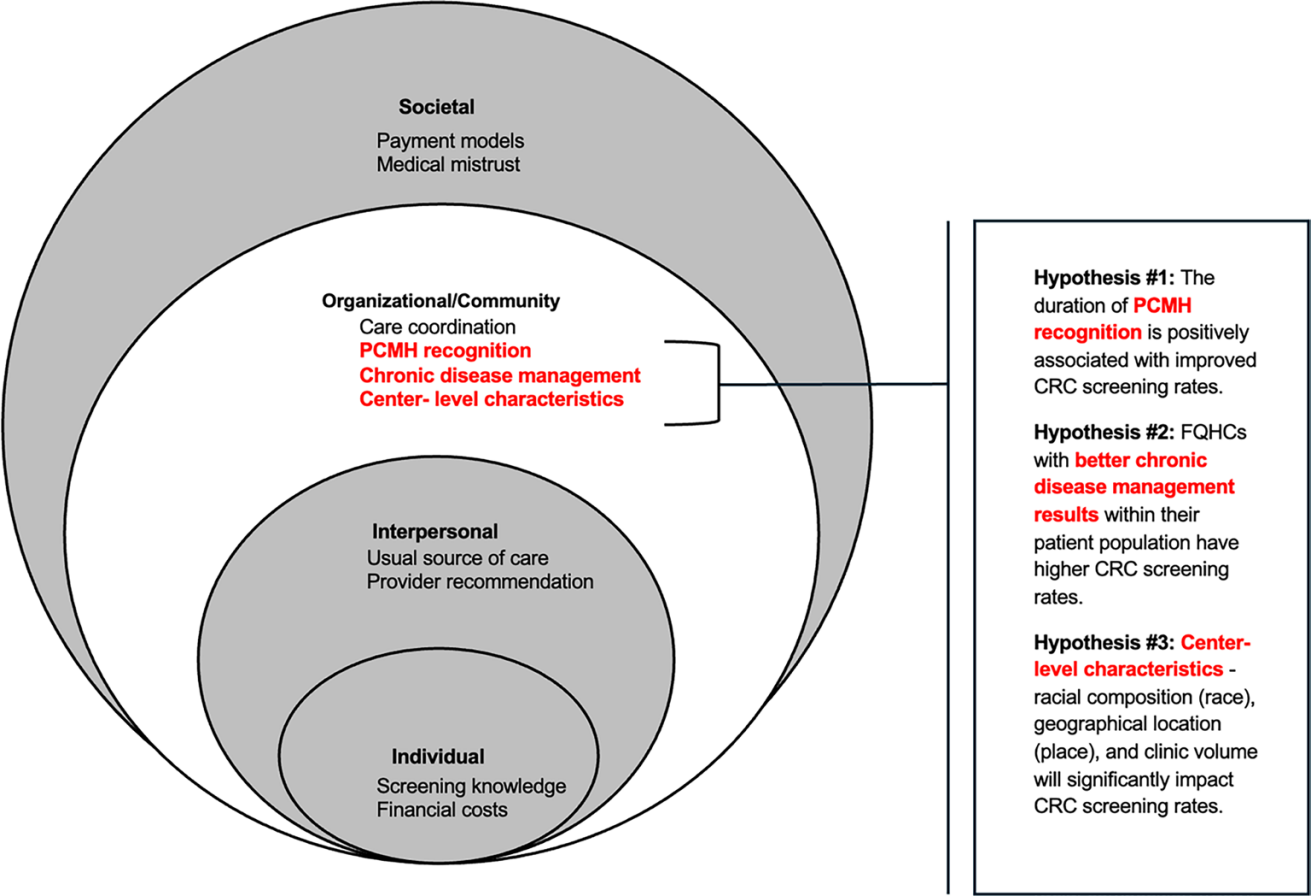
Socioecological factors of CRC screening uptake in FQHCs. Conceptual model of socioecological factors that impact colorectal cancer screening uptake in FQHCs, adapted from the socioecological model. New factors analyzed in this study are bold in red font.

This study examined trends in CRC screening rates across FQHCs in the US, identifying key organizational‐level factors such as PCMH recognition and duration, chronic disease management, and center characteristics. Figure 1 is a visual depiction of both known and newly established factors that are associated with CRC screening rates. Presented within the concentric circles of the socioecological system that influences health outcomes [20, 21], the new factors at the organizational level are presented alongside known predictors at the individual, interpersonal, and systemic levels. This figure clarifies the contribution of this study, while also highlighting the need for multi‐level interventions to influence screening behaviors in historically marginalized communities.

By addressing systemic barriers and improving operational efficiency, these findings offer actionable strategies to enhance cancer screening delivery. The study aimed to track changes in CRC screening rates over time and determine organizational‐level factors influencing these shifts. We proposed three hypotheses to guide the analysis: (1) the duration of PCMH recognition is positively associated with improved CRC screening rates, (2) FQHCs with better chronic disease management results within their patient population have higher CRC screening rates, and (3) center‐level characteristics—racial composition (race), geographical location (location), and center volume (size)—will significantly impact CRC screening rates.

## 2 Methods

This study analyzed Uniform Data System (UDS) data from 2017 to 2022, capturing trends in FQHCs including time during the COVID‐19 pandemic. Managed by the Health Resources and Services Administration (HRSA), the UDS provides standardized annual reports on operational, financial, and clinical outcomes. Data were aggregated at the organizational level, with FQHC participation ranging from 1361 to 1385 centers. The dataset includes administrative variables such as patient demographics, insurance sources, and center characteristics, alongside clinical measures like quality metrics and screening rates. This study included FQHCs receiving full funding from the Public Health Service Section 330 grants, located within the 50 states/D.C., and reported CRC screening measures. School‐based centers, FQHCs located in US territories, and look‐alike centers which do not receive full funding were excluded.

### 2.1 Measures

All measures were taken from the UDS and are defined below (full variable details can be found in Appendix A) [22].

The primary outcome is *CRC screening*, defined by the UDS, as the percentage of eligible adults aged 50 to 75 who had a medical visit and received an appropriate CRC screening, as recommended by the United States Preventive Services Task Force (USPSTF). Exclusions from the measure include individuals diagnosed with CRC, with a history of total colectomy, in hospice care, aged 66 and older living in long‐term care for more than 90 days, and aged 66 and older with advanced illness and frailty. Appropriate screenings include: fecal occult blood test (FOBT) during the measurement period; fecal immunochemical test (FIT)‐DNA during the measurement period or the two years prior; flexible sigmoidoscopy or computerized tomography (CT) colonography during the measurement period or the four years prior; and colonoscopy during the measurement period or the nine years prior. The key independent variables are listed below.

*Patient‐Centered Medical Home (PCMH)* recognition. This is a dichotomous variable (yes/no) recoded to measure length in PCMH during the study period (none, at least 1 year, all years).

*Chronic Disease Management (CDM)*. Chronic disease management focused on two continuous health outcomes: uncontrolled diabetes (inverse measure) and controlled hypertension. We used the Million Hearts [23] and *HealthyPeople2030* [24] guidelines (controlled hypertension ≥ 80% and uncontrolled diabetes < 11.6% in the patient population). Uncontrolled diabetes is defined as the percentage of patients aged 18–75 with a diabetes diagnosis who had a hemoglobin A1c (HbA1c) greater than 9%. Controlled hypertension is defined as the percentage of patients aged 18–85 with a hypertension diagnosis whose most recent blood pressure reading was less than 140/90.

*Black Patient Population*. The percentage of Black patients (self‐reported as Black or African American) receiving care in the FQHC is categorized into three levels: below 10%, 10%–20%, and greater than 20% of the total patient population. These thresholds reflect distribution frequencies across the dataset and were anchored around the national average of 19%.

Covariates of interest were the location and size (total patient population) of the FQHC, percentage of patients who were age eligible for screening, and distribution of Medicare and Medicaid insurance coverage in the population. This study received approval from The Ohio State University Institutional Review Board (Protocol #2023E1185) and classified as a secondary data analysis of deidentified publicly available data.

## 3 Statistical Analysis

All analyses were conducted using Stata 18 [25]. We generated descriptive statistics to summarize the data, reporting continuous variables as means with standard deviations and categorical variables as frequencies and percentages. Bivariate analyses with chi‐square testing were conducted to examine differences in categorical variables across the six‐year study period. Continuous variables exhibiting non‐normality, skew, and inflation around specific values were transformed into categorical variables to enhance model fit and improve interpretability in graphical presentations. Variables that demonstrated statistical and/or theoretical significance were then explored further using mixed effects models. The findings from the mixed effects models are reported in this study.

Multivariate mixed‐effects models, as outlined by O'Connell et al. [26], were used to assess associations between CRC screening rates and key independent variables, accounting for covariates, temporal changes, and data hierarchy. Fixed effects included specific organizational and population attributes such as PCMH recognition, management of hypertension and diabetes, distribution of Black patients, insurance status, size, and location. Random effects accounted for variability at the FQHC level to capture differences between organizations.

The multilevel model had two levels: level 1 captured within‐FQHC variability over time, and level 2 captured between‐FQHC variability in the level 1 growth parameters. After graphing individual growth curves, both linear and nonlinear models were developed. The growth curves indicated nonlinearity, analogous to baseline estimates using UDS data from 2017 to 2021, showing distinct growth phases. Due to nonlinearity in the outcome, a piecewise model was used, with time recoded into three distinct time pieces to capture varying growth rates across study periods. Maximum likelihood estimation was used to test and compare contributions of level 2 variables in the statistical model.

An unconditional linear growth model was analyzed to predict the initial level and average growth trajectory across FQHCs. The intraclass correlation coefficient (ICC) was calculated to measure the variance in CRC screening rates between FQHCs. To make time meaningful at the intercept, the time variable was centered around the first year of the study. Level 2 variables were added individually to evaluate their contribution to CRC screening uptake, significance, and model fit. We used the margins command to estimate predicted values and examine interaction effects across variables and time periods. The best‐fitting model, selected based on fit statistics, determined the variability in screening uptake across the study period. All statistical measurements were two‐sided with a significance level of 0.05. Optimal Design software assessed the study design, showing a minimal detectable effect size of 0.20 with 80% power.

## 4 Results

**FIGURE 2:**
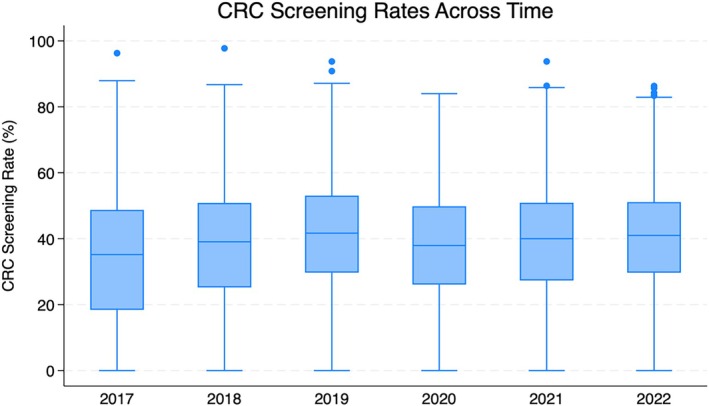
Box and whisker plot depicting CRC screening rates from 2017 to 2022, illustrating the median, interquartile range, and outliers for each year.

Our final sample included 7692 observations from 1282 FQHC organizations. Table 1 compares the characteristics of FQHCs by year. The number of delivery sites per FQHC varied substantially, with most averaging between 8 and 11 sites, while the largest reported 162. Thirty‐four percent of FQHCs were in the Southern US, primarily in urban areas. Over the study period, FQHCs served an average of 27.8 million patients, with nearly 70% being racial and/or ethnic minorities. The average CRC screening rate ranged from 33% to 40% over the six years, regardless of time (Figure 2).

**TABLE 1:** Sample characteristics of FQHCs across six years.

Variables	*n* = 1282 FQHCs					
	2017 *M(SD) or %*	2018 *M(SD) or %*	2019 *M(SD) or %*	2020 *M(SD) or %*	2021 *M(SD) or %*	2022 *M(SD) or %*
#Delivery sites per FQHC	8.29 (9.17)	8.92 (9.96)	9.62 (11.03)	10.26 (11.65)	10.84 (12.09)	11.40 (12.90)
	*min = 1*	*min = 1*	*min = 1*	*min = 1*	*min = 1*	*min = 1*
	*max = 105*	*max = 111*	*max = 135*	*max = 139*	*max = 154*	*max = 162*
Community type						
Rural	40.34%	42%	39.05%	38.99%	39.21%	39.75%
Urban	59.66%	58%	60.95%	61.01%	60.79%	60.25%
Total #Patients (millions)	26	27.2	28.4	27.4	29	29.2
CRCS rate	33.39	37.82	40.86	37.63	38.93	40.37
	(20.68)	(19.10)	(18.07)	(7.25)	(17.32)	(16.06)
Race/Ethnicity						
Black	19.88	19.74	19.46	19.31	19.37	19.12
	(23.59)	(23.31)	(22.97)	(22.99)	(23)	(22.80)
White	59.01	58.83	58.62	58.59	58.00	57.42
	(28.89)	(28.38)	(28.12)	(27.74)	(27.69)	(27.60)
Hispanic	25.73	26.01	26.38	26.02	26.62	27.45
	(25.74)	(25.52)	(25.50)	(25.39)	(25.49)	(25.73)
Non‐Hispanic	70.98	70.51	69.77	69.77	68.62	67.57
	(25.95)	(25.84)	(25.75)	(26.14)	(25.78)	(26.09)
Language other than English preferred	17.60	17.31	18.10	18.05	18.28	19.21
	(20.70)	(19.71)	(19.87)	(19.74)	(19.90)	(20.41)
Black patient population > 20% Yes	35.26	35.73	36.27	36.12	36.43	35.65
Age eligible patients						
50–75	26.89	27.16	27.43	28.78	28.80	28.90
	(8.61)	(8.54)	(8.57)	(8.50)	(8.47)	(8.47)
FPL < 100%	47	46.24	45.81	44.17	42.53	42.53
	(23.25)	(22.82)	(22.66)	(22.89)	(22.73)	(22.98)

Variables	2017	2018	2019	2020	2021	2022
	*M(SD) or %*	*M(SD) or %*	*M(SD) or %*	*M(SD) or %*	*M(SD) or %*	*M(SD) or %*
Insurance/Payors						
Medicaid	42.93	42.52	41.77	41.13	42.38	44.51
	(19.43)	(18.98)	(18.50)	(17.99)	(18.01)	(18.16)
Medicare	10.69	11.05	11.21	11.88	11.99	12.26
	(7.07)	(7.25)	(7.25)	(7.44)	(7.43)	(7.65)
Dual eligible	3.96	3.95	3.92	4.12	4.19	4.36
	(2.79)	(2.81)	(2.85)	(3.01)	(3.01)	(3.13)
Private	19.85	20.32	20.95	22.13	21.90	21.62
	(12.86)	(12.76)	(12.92)	(13.01)	(12.73)	(12.55)
Uninsured	25.18	24.83	24.89	23.70	22.37	20.61
	(17.94)	(17.55)	(17.57)	(17.13)	(16.89)	(16.54)
Chronic disease management						
DM	31.55	32.32	31.02	34.39	31.41	29.66
	(13.60)	(11.68)	(11.00)	(11.26)	(10.63)	(9.17)
HTN	58.06	60.68	62.61	56.45	58.54	62.15
	(16.89)	(13.58)	(12.46)	(11.81)	(12.08)	(10.23)
PCMH Yes	78.16%	NR	NR	79.13%	78.55%	78.81%

Abbreviations: CRCS, colorectral cancer screening; DM, percentage of uncontrolled diabetes in the population; FPL, federal poverty level (below 100%); HTN, percentage of controlled hypertension in the population; NR, Not Reported; PCMH, Patient‐Centered Medical Home.

Key factors—including the duration of PCMH recognition, proportion of controlled hypertension, racial composition, geographic location, and the size of FQHCs—were associated with variations in CRC screening rates, as illustrated in Figures B1–B5. Time was measured across three periods: 2017–2019; piece 1, 2019–2020; piece 2, and 2021–2022; piece 3. Overall, CRC screening rates were on an upward trend, experiencing a significant decline between 2019 and 2020. Rates have not fully recovered to levels in time piece 1 (Figure 2). Table 2 presents both the adjusted and unadjusted main effects results from the mixed‐effects models.

Hypothesis 1 *The duration of PCMH recognition is positively associated with improved CRC screening rates*.

**TABLE 2:** Main effects results from unadjusted and adjusted models.

Variable	Unadjusted coefficient (95% CI)	Adjusted coefficient (95% CI)	*p*‐value***
PCMH years			
None	Ref	Ref	
At least 1	9.79 (5.987, 13.59)**	5.70 (2.27, 9.13)**	0.001
All	14.37 (11.08, 17.65)**	8.50 (5.44, 11.57)**	0.000
Controlled hypertension	0.37 (0.33, 0.41)**	0.35 (0.31, 0.39)**	0.000
Uncontrolled diabetes	0.13 (0.09, 0.18)**	0.02 (−0.03, 0.07)	0.82
Proportion of black patients			
< 10%	Ref	Ref	
10%–20%	−4.13 (−6.66, −1.60)**	−3.24 (−5.72, −0.77)*	0.010
> 20%	−4.65 (−6.85, −2.44)**	−2.98 (−5.25, −0.70)*	0.010
Size			
Small	Ref	Ref	
Medium	2.67 (0.56, 4.79)*	1.66 (−0.39, 3.71)	0.112
Large	4.04 (1.47, 6.61)**	2.68 (0.11, 5.24)*	0.041
Region			
Northeast	Ref	Ref	
Midwest	−5.63 (−9.25, −2.02)**	−5.40 (−8.58, −2.21)**	0.001
South	−9.42 (−12.66, −6.18)**	−5.95 (−8.91, −3.00)**	0.000
West	−5.50 (−8.50, −2.13)**	−4.70 (−7.82, −1.58)**	0.003
Medicare	0.27 (0.13, 0.40)**	0.27 (0.11, 0.43)**	0.001
Medicaid			
Bottom 25th	Ref	Ref	
25th–75th	2.39 (0.49, 4.20) *	1.0 (−0.87, 2.87)	0.293
Top 75th	1.91 (−0.35, 4.17)	−0.18 (−2.28, 2.21)	0.881
Age eligible population	−0.07 (−0.18, 0.05)	−0.11 (−0.25, 0.03)	0.136

*Note:* * *p* < 0.05; ** *p* < 0.005; Ref = reference group; *** *p*‐value from adjusted model. Interaction effects capturing time‐varying associations are omitted for brevity. Complete model outputs, including interaction terms, are available in Appendix B.

The duration of PCMH recognition emerged as the most significant predictor of CRC screening rates. Consistently, more than three‐quarters of FQHCs were recognized as a PCMH across the study period. FQHCs with at least one year of PCMH recognition had a significant increase in CRC screening rates (*β* = 5.70, *p* < 0.01) compared to those without PCMH recognition. Additionally, centers with consistent PCMH recognition across all reported years showed an even greater increase in CRC screening rates (*β* = 8.50, *p* < 0.001).

Hypothesis 2 *FQHCs with better chronic disease management results within their patient population have higher CRC screening rates*.

Across the six‐year study period, only 3.3% of FQHCs met the Healthy People 2030 goal of maintaining uncontrolled diabetes (Hgb A1C > 9) below 11.6% of their patient population with diabetes. On average, 31.8% of patients across all FQHCs had uncontrolled diabetes. Similarly, only 2.9% of FQHCs met the Million Hearts goal of maintaining blood pressure control (BP < 140/90) in at least 80% of their patient population with hypertension. The average rate of controlled hypertension across the study period was 59.8%. Having a higher proportion of patients with controlled hypertension was significantly associated with increased CRC screening rates (*β* = 0.354, *p* < 0.0001). The overall effect of uncontrolled diabetes was not statistically significant in the adjusted model (Table 2). However, there were time‐varying effects, with larger populations with uncontrolled diabtes who demonstrated significant reductions in CRC screening rates in the 2017–2019 (*β* = −0.04, *p* = 0.019) and 2021–2022 (*β* = −0.06, *p* = 0.001) time periods.

Hypothesis 3 *Center‐level characteristics—racial composition (race), geographical location (place), and center volume (size) will significantly impact CRC screening rates*.

Compared to FQHCs with fewer than 10% of Black patients, those with 10%–20% (*β* = −3.24, *p* = 0.010) and greater than 20% (*β* = −2.98, *p* = 0.010) of Black patients showed a negative association with CRC screening rates, with no significant time‐varying effects observed. There were significant regional variations in CRC screening rates, with the Northeast region showing the highest rates overall, while FQHCs in the Southern region had the lowest rates, with a predicted 5.95 percentage point decrease in screening rates compared to those in the Northeast. Relative to Northeastern FQHCs, only those in the South experienced a significant increase in CRC screening rates by 3.9 points in time period 1 (Wald's z = 2.19, *p* = 0.028); however, this growth was not sustained during time period 3. Compared to small FQHCs, large organizations had the most significant rate of decline in CRC screening rates during the second time period, decreasing by 1.35 points (Wald's z = −2.56, *p* = 0.010). Despite this, large FQHCs showed the fastest recovery during the third time period, with screening rates increasing by 2.46 points (Wald's z = 2.32, *p* = 0.020). Notably, there were time‐varying effects across regions and sizes of FQHCs (Figure 2). Full mixed effects model results are available in Table B1.

We used mixed‐effects models with interaction terms and post‐estimation margins to estimate predicted CRC screening rates across levels of PCMH recognition and key covariates (Table B2). The presence and duration of PCMH recognition had a positive impact on CRC screening rates across all variables. Among FQHCs with the highest burden of uncontrolled diabetes, predicted CRC screening rates were 24.05% in those with no PCMH recognition, compared to 36.12% in FQHCs with recognition across all years—a 12.07 percentage point increase. FQHCs with at least one year of PCMH recognition saw a 7.73‐point improvement.

At FQHCs with the lowest levels of controlled hypertension, predicted CRC screening rates were 27.52% in sites without PCMH recognition. FQHCs with at least one year of recognition had rates of 32.99%, and those with recognition across all years reached 36.37%, reflecting a 5.47‐point and 8.85‐point increase, respectively. At FQHCs where the population of Black patients exceeded 20% (greater than national FQHC average), predicted CRC screening rates were 27.28% in those without PCMH recognition. FQHCs with at least one year of recognition reached 35.26%, and those with recognition across all years achieved 39.48%, reflecting an increase of 7.98 and 12.21 percentage points, respectively.

In the Southern region—where CRC screening rates were consistently lowest—FQHCs without PCMH recognition had a predicted screening rate of 29.94%. Rates rose to 34.57% among those with at least one year of recognition, and to 37.38% for FQHCs with recognition across all years. This reflects a 4.63‐point increase for partial recognition and a 7.44‐point increase for sustained recognition. Smaller FQHCs consistently exhibited lower predicted CRC screening rates across PCMH recognition levels, with rates rising from 31.27% (without PCMH) to 38.71% (PCMH all years). By comparison, large FQHCs reached 40.39% with full recognition, reflecting a 7.44‐point gap between small and large FQHCs.

## 5 Discussion

Our study examined changes in CRC screening rates over time in FQHCs and identified organizational‐level factors which may impact screening uptake. The results are consistent with our hypotheses, suggesting that the duration of PCMH recognition, having better chronic disease management (i.e., hypertension), and center‐level characteristics—such as racial composition, location, and volume/size—are significantly associated with CRC screening rates in FQHCs. However, our hypothesis regarding chronic disease management for uncontrolled diabetes was not confirmed, as it lost statistical significance in the adjusted model. This may reflect the complexity of diabetes management, which often requires more frequent touchpoints, multifaceted interventions, and sustained patient engagement than CRC screening. The poor diabetes management performance observed in our FQHC sample may indicate that these strategies have not yet been fully mastered or optimized, potentially limiting their spillover impact on preventive services like CRC screening. Our results should be interpreted with caution, given the potential for unobserved confounding and limitations in capturing patient‐ and delivery site‐level variables that may influence outcomes.

Overall, PCMH recognition is positively associated with cancer screening uptake. A retrospective study utilizing the UDS revealed that FQHCs with at least one delivery site recognized as a PCMH demonstrated significantly higher CRC screening rates compared to FQHCs without any PCMH‐recognized delivery sites [16]. Our study builds on previous research and offers suggestive evidence that long‐term implementation of PCMH may support improvements in CRC screening outcomes. Importantly, this positive association persists even in the face of substantial challenges such as suboptimal chronic disease management and global health crises like the COVID‐19 pandemic, factors that uniquely shaped care delivery during the study period.

Notably, predicted probabilities were elevated among PCMH‐recognized FQHCs serving a large percentage of Black patients, pointing to a potential role for structured care models in supporting preventive services in communities facing barriers that may include socioeconomic disadvantage, chronic disease burden, and limited English proficiency [27]. These findings suggest that sustained PCMH models may contribute to reducing disparities and supporting cancer screening efforts. While the body of literature on the longitudinal effects of PCMH recognition on health outcomes in general remains limited, Maeng et al. found that practices with longer PCMH exposure had fewer patients with acute hospitalizations [28]. Likewise, a study examining PCMH duration and quality of care noted a 19.1 percentage point increase in the receipt of guideline‐concordant care when practices were in PCMH for at least a year, compared to one‐month [29].

While our study found that the duration of PCMH recognition is a significant factor of CRC screening uptake, it remains unclear which specific components of the PCMH model contribute most to these improved outcomes. The National Committee for Quality Assurance recognizes PCMH based on six components, 40 core criteria, and 25 elective criteria [30]. However, our study could only ascertain whether a FQHC had PCMH recognition and for how long, without the ability to analyze performance across these individual components. Therefore, further research is essential to delineate which aspects of the PCMH model drive the greatest improvements in cancer screening uptake to focus practice facilitation efforts.

The proportion of controlled hypertension in the population is significantly associated with CRC screening. This may reflect the strength of FQHCs in delivering coordinated, team‐based care, particularly for patients with chronic conditions like hypertension that have been associated with increased poor health outcomes and cancer risk [31, 32]. These strategies, such as patient education, patient navigation, referral management, using patient tracking and alerts, staff training, and EHR registries, are crucial for effective chronic disease management, often delivered by non‐provider care team members. This reaffirms the appropriateness of an interdisciplinary team approach to cancer screening [33, 34].

It is plausible that FQHCs apply these same strategies across their patient population, which could explain the observed association. Integrating population health management strategies with CRC screening emphasizes a proactive, programmatic approach focused on preventive health for the patient population, rather than relying solely on opportunistic screenings during office visits [35]. By utilizing a consistent, comprehensive approach, FQHCs can improve hypertension outcomes and CRC screening rates, demonstrating the effectiveness of population health strategies across multiple health conditions. Although uncontrolled diabetes was not significantly associated with CRC screening in adjusted models, time‐varying effects may reflect shifting priorities, care adaptations, or residual confounding, especially within FQHCs, where diabetes control remained consistently low.

Race and location of care have long been critical determinants of health outcomes [36]. Our findings confirmed this for CRC screening as well. FQHCs with higher proportions of Black patients had significantly lower screening rates than those with fewer Black patients. FQHCs in the southern region, where the majority of Black Americans reside [37], had the lowest screening rates. This region includes states that did not expand Medicaid and have consistently ranked among the lowest in healthcare quality [38]. Expanding Medicaid coverage improves access to and utilization of preventive services, which can help mitigate these inequities and enhance overall health outcomes [38]. These disparities highlight the impact of structural racism on healthcare, with significant effects on patient outcomes due to differences in care locations and resource allocation. Recognizing that Black Americans have the highest mortality rate from CRC, FQHCs must commit to culturally responsive outreach and messaging. By collaborating with community partners, they can work to eliminate barriers to screening and improve CRC outcomes [39]. For example, by bundling cancer screening with social needs screening through partnerships with food and housing organizations, which has been shown to increase screening uptake in underserved populations [40].

Addressing these inequities is essential to improving health outcomes and ensuring equitable access to preventive care for all populations. Additionally, the volume/size of FQHCs plays a significant role in screening rates, with larger FQHCs showing faster recovery in screening rates compared to those of smaller and mid‐size. Connecting smaller FQHCs with larger centers can be beneficial, as larger FQHCs may have more resources and best practices that can support smaller sites in improving their clinical operations and patient care. Strengthening the use of quality improvement coalitions through existing primary care associations, where performance metrics can be tracked and monitored and FQHCs can partner for resource sharing and best practices, further enhances these efforts. Addressing these inequities is essential to improving health outcomes and ensuring equitable access to preventive care for all populations.

## 6 Policy and Practice Implications

Policies supporting the adoption and maintenance of PCMH recognition should be advocated to ensure that more FQHCs can implement and sustain these comprehensive care models. Additionally, providing resources and support for ongoing training and development within PCMH‐recognized FQHCs could further bolster their effectiveness in improving health outcomes. Coupling chronic disease management with CRC screening, by embedding standardized strategies such as team‐based care, patient registries, and navigation support, may help improve screening uptake while reinforcing overall health outcomes. By prioritizing these integrated care strategies, FQHCs can effectively address chronic disease management while boosting CRC screening uptake.

## 7 Strengths and Limitations

Our study's strengths include a large sample size and comprehensive UDS data from HRSA, enhancing generalizability and enabling analysis of key predictors, including understudied factors in this population. Limitations include reliance on organizational‐level data, which may mask site‐level variation; unmeasured confounders such as patient education and income; potential reporting bias due to pooled delivery‐site data and electronic health record constraints; variability and missingness in reported metrics across FQHCs, which may limit consistency across sites; and disruptions related to the COVID‐19 pandemic. While the pandemic significantly affected CRC screening rates, primarily through work stoppages and resource reallocation as noted in prior studies, we opted not to focus on this well‐documented influence. Instead, we concentrated on structural and organizational predictors, while acknowledging the importance of pandemic‐related disruptions as contextual factors. To address these challenges, mixed‐effects models account for site‐level differences and hierarchical structures, while longitudinal data from 2017 to 2022 contextualizes trends and recovery post‐pandemic. These methods strengthen the study's ability to provide reliable insights into CRC screening disparities in FQHCs.

## 8 Conclusion

This study highlights the potential of organizational‐level approaches to improve CRC screening rates in FQHCs. While models like PCMH may support screening delivery through collaboration, efficiency, and patient‐centered care, their impact remains context‐dependent and may be influenced by unobserved factors. Integrating chronic disease management into broader initiatives may contribute to long‐term cancer prevention. Smaller FQHCs could benefit from strategic resource‐sharing with larger centers, though such collaborations may be shaped by competitive dynamics and funding constraints. These findings suggest that organizational innovations, when attuned to local conditions, may help advance health equity and expand access to cancer screening for underserved populations.

## Funding

This work was supported by the National Cancer Institute (T32‐CA‐236621).

## Conflicts of Interest

The authors declare no conflicts of interest.

## Supporting information


**Appendix A.** Study variables details.
**Appendix B**. CRC screening means, predicted margins, and full mixed effects results.
**Figure B1**. CRC screening trends by duration of PCMH recognition.
**Figure B2**. CRC screening trends by hypertension control.
**Figure B3**. CRC screening trends by proportion of black patients.
**Figure B4**. CRC screening trends by regional differences.
**Figure B5**. CRC screening trends by and FQHC size.
**Table B1**. Mixed effects model: fully adjusted with main and interaction effects.
**Table B2**. Adjusted margins: CRC screening rates by PCMH recognition across representative thresholds of organizational‐level variables.

## Data Availability

The data that support the findings of this study are available in Uniform Data System at https://www.hrsa.gov/foia/electronic‐reading. These data were derived from the following resources available in the public domain: Uniform Data System, https://www.hrsa.gov/foia/electronic‐reading.
