# Supplementary material for: Predictors of Colorectal Cancer Screening Rates in Federally Qualified Health Centers: Explicating Organizational Level Factors
Source: Health Serv Res. 2026 Jan 4;61(2):e70082. doi: 10.1111/1475-6773.70082 (PMC12932028; doi:10.1111/1475-6773.70082)
Supplement: Supplementary file 1 — Appendix A. Study variables details. Appendix B. CRC screening means, predicted margins, and full mixed effects results. Figure B1. CRC screening trends by duration of PCMH recognition. Figure B2. CRC screening trends by hypertension control. Figure B3. CRC screening trends by proportion of black patients. Figure B4. CRC screening trends by regional differences. Figure B5. CRC screening trends by and FQHC size. Table B1. Mixed effects model: fully adjusted with main and interaction effects. Table B2. Adjusted margins: CRC screening rates by PCMH recognition across representative thresholds of organizational‐level variables. [file HESR-61-e70082-s001.docx]

Appendix A: Study Variables Details

| Table A1. Full Study Variables Details: Description, Transformation, and Rationale | | | |
| --- | --- | --- | --- |
| Variable | Description | Encoding | Rationale |
| Outcome Measure | | | |
| Colorectal cancer Screening | Percentage of age-eligible adults up to date with USPSTF approved screening. | Continuous: no transformation | Outcome Variable |
| Primary Predictors | | | |
| Patient Centered Medical Home | A patient-centered model of care in which Federally Qualified Health Centers (FQHCs) may receive formal recognition from The Joint Commission or the National Committee for Quality Assurance. The Uniform Data System (UDS) reports PCMH status annually, though data were not available for 2018 and 2019. | 0 = None  1 = At least 1 year  2 = All years reported | Originally coded dichotomously (yes/no), this variable was recoded into three categories reflecting temporal exposure.  Categorization accounts for variation in PCMH implementation across FQHCs and improves model fit. It also enhances interpretability by capturing trends in duration of exposure to PCMH recognition, aligning with analyses of longitudinal care delivery models. |
| Chronic Disease Management (Uncontrolled Diabetes/Controlled Hypertension) | As reported in the Uniform Data System (UDS), hypertension control is defined as blood pressure <140/90 mmHg. Diabetes control is captured using an inverse metric—Hemoglobin A1c >9%—indicating poor glycemic control. | 0 = Least controlled: Below the 25^th^ percentile and below  1 = Less controlled: 25^th^-49^th^ percentile  2 = More controlled: 50^th^-75^th^ percentile  3 = Most controlled: Above the 75^th^ percentile  **Note**: For diabetes, quartile interpretation was inverse—higher percentiles reflect poorer control, indicating a greater proportion of the population with uncontrolled diabetes (Hemoglobin A1c >9%). | **Diabetes and hypertension are HRSA-priority clinical quality metrics frequently paired in population health management efforts.**  This measure, originally reported as a continuous percentage, was transformed into a categorical variable with four levels based on natural quartile distributions for graphical display.  Continuous chronic disease management variables were transformed into quartiles to improve interpretability and highlight meaningful performance differences across health centers. Non-normal distributions limited parametric utility and masked underlying patterns; categorization enabled alignment with common quality benchmarks and clearer communication for clinical and policy audiences. |
| Black patient population | The percentage of patients who self-reported their race as Black or African American. | 0 = Small population: <10%  1 = Average population: 10-20%  2 = Large population: >20% | This measure, originally reported as a continuous percentage, was transformed into a categorical variable with 3 levels based on the national average of Black patients served across all FQHCs (~19%).  This measure was transformed into a categorical variable to address its non-normal distribution and enhance interpretability. Significant variation in outcomes was observed at different levels of the proportion of Black patients, which would likely be obscured if modeled continuously. Categorizing the measure using meaningful cut points—anchored to the national average within the FQHC population (~19%)—enabled clearer comparisons across health centers and surfaced threshold effects. |
| Covariates | | | |
| Location | Location is defined as the US region a FQHC was located (North, South, East, West) | 1 = Northeast  2 = Midwest  3 = South  4 = West | Geographic disparities in colorectal cancer screening uptake are well documented, with screening rates varying significantly across U.S. regions. Grouping states into CDC-defined regions enables exploration of structural, policy, and resource-based differences that may contribute to inequities in access and utilization.  Region was derived from a nominal state-level variable. Using CDC-assigned geographic classifications, states were numerically coded (e.g., AK = 1, AL = 2, AR = 3…) and grouped into regions to create a categorical variable reflecting geographic location. |
| FQHC size | The number of unique patients reported being served each year. | **1 = Small:** Below the 25^th^ percentile  **2 = Mid-sized:** 25^th^ to 74^th^ percentile  **3 = Large:** 75^th^ percentile and above | The Uniform Data System (UDS) reports the total number of patients served annually by each FQHC. We derived a new variable to classify FQHCs by size, based on their reported patient population across study years. Using the mean patient count as a reference, health centers were grouped into three categories.  Health center size has been associated with meaningful variation in colorectal cancer screening performance. The measure was not normally distributed and modeling it as continuous risked masking important outcome differences.  By categorizing centers based on patient population tiers, we captured granular distinctions in operational scale and clinical capacity. This approach adds interpretive nuance and enables more targeted comparisons—particularly in examining how resource allocation and infrastructure may influence screening uptake. |
| Age-Eligibility | The UDS reports CRC screening for adults who are 50-75 years of age on 6/30^th^ of the reporting year. | **0 = Low proportion:** Below the 25^th^ percentile  **1 = Moderate proportion:** 25^th^ -75^th^ percentile  **2 = High proportion:** Above the 75^th^ percentile | Age has been consistently associated with increased CRC screening uptake. In the absence of patient-level data, we used the proportion of age-eligible patients served by each FQHC to adjust for demographic differences across sites. This approach accounts for health centers with higher concentrations of older adults who may be more likely to engage in screening, improving the accuracy of performance comparisons across populations.  We calculated the percentage of age-eligible patients (aged 50–74; as reported by the UDS) served by each FQHC in each reporting year. This continuous variable was then recoded into three categorical levels based on natural quartile distribution. |
| Insurance Coverage | The number of patients for each FQHC receiving each type of insurance. | **0 = Low proportion:** Below the 25^th^ percentile  **1 = Moderate proportion:** 25^th^ -75^th^ percentile  **2 = High proportion:** Above the 75^th^ percentile | Insurance status is a well-established determinant of CRC screening uptake. To account for potential differences related to public insurance coverage, we examined the proportion of Medicare and Medicaid enrollees within each FQHC population. Given the measure’s non-normal distribution and the presence of meaningful outcome variation across coverage levels, we transformed it into a three-level categorical variable. This approach improves interpretability with graphical display and allows for nuanced comparison across payer mix strata that may influence access to screening services.  The original variable reported raw counts of Medicare and Medicaid enrollees. We created a new variable representing the percentage of these enrollees within the total patient population. This continuous percentage variable was then recoded into three categorical levels based on its natural quartile distribution. |

Appendix B: CRC Screening Means, Predicted Margins, and Full Mixed Effects Results

**Figure B1: CRC Screening Trends by Duration of PCMH Recognition**

**
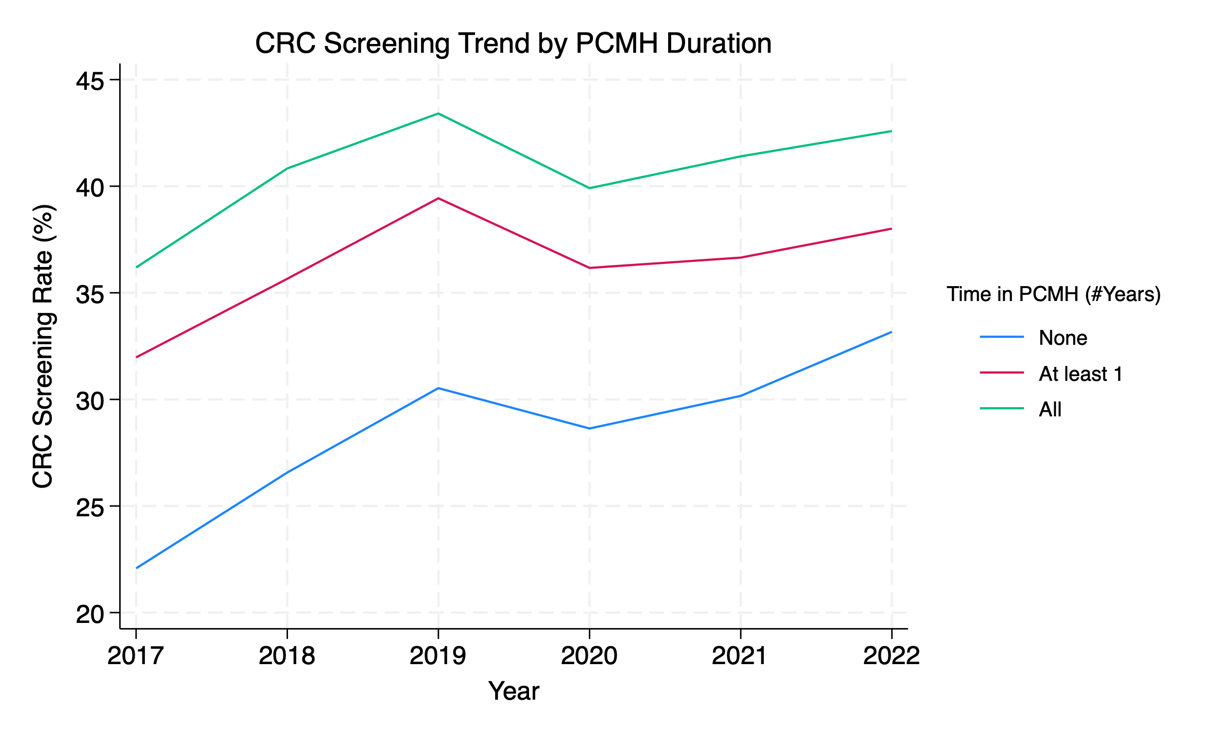
**

Note: Line graphs showing trends in CRC screening rates from 2017 to 2022, stratified by PCMH duration.

**Figure B2: CRC Screening Trends by Hypertension Control**

**
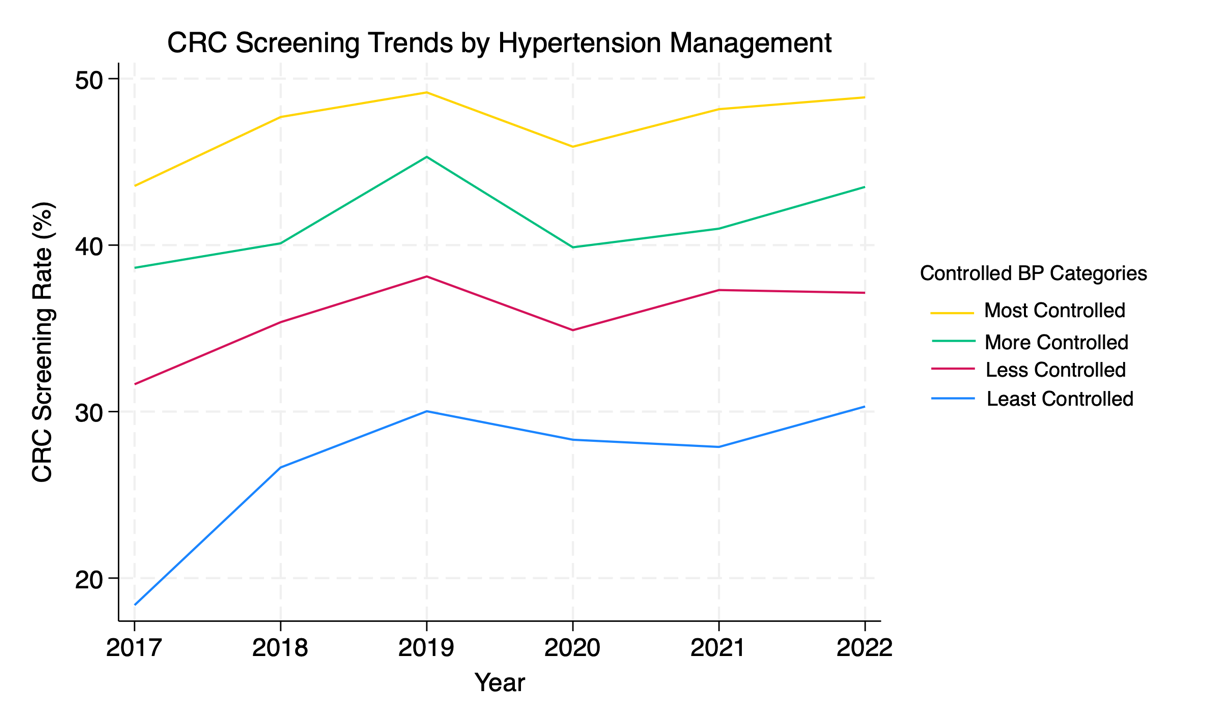
**

Note: Line graphs showing trends in CRC screening rates from 2017 to 2022, stratified by hypertension management.

**Figure B3: CRC Screening Trends by Proportion of Black Patients**

**
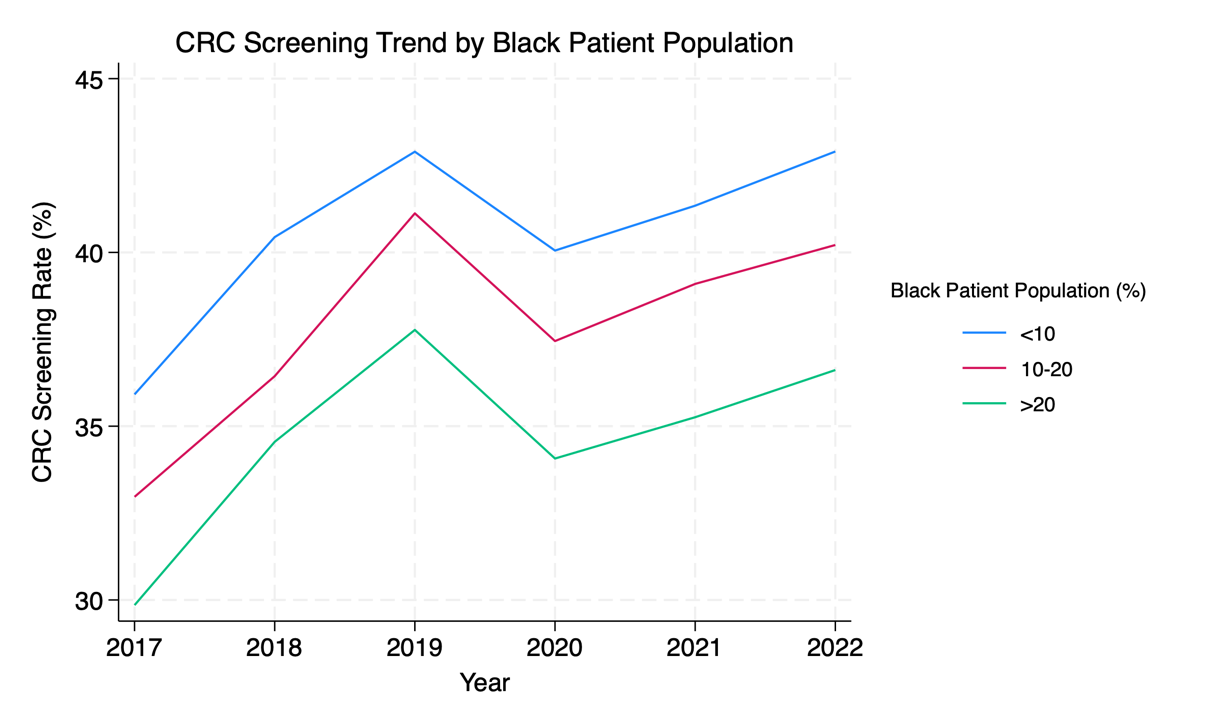
**

Note: Line graphs showing trends in CRC screening rates from 2017 to 2022, stratified by Black patient population percentage.

**Figure B4: CRC Screening Trends by Regional Differences**

**
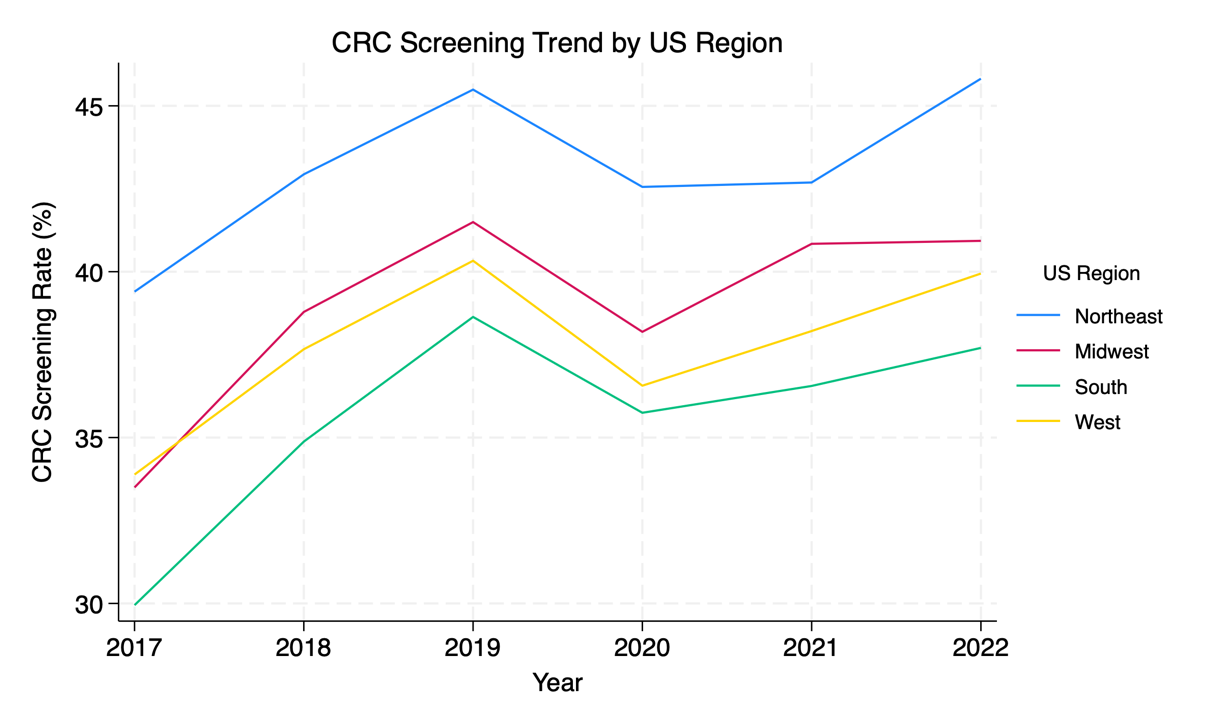
**

Note: Line graphs showing trends in CRC screening rates from 2017 to 2022, stratified by US region.

**Figure B5: CRC Screening Trends by and FQHC Size**

**
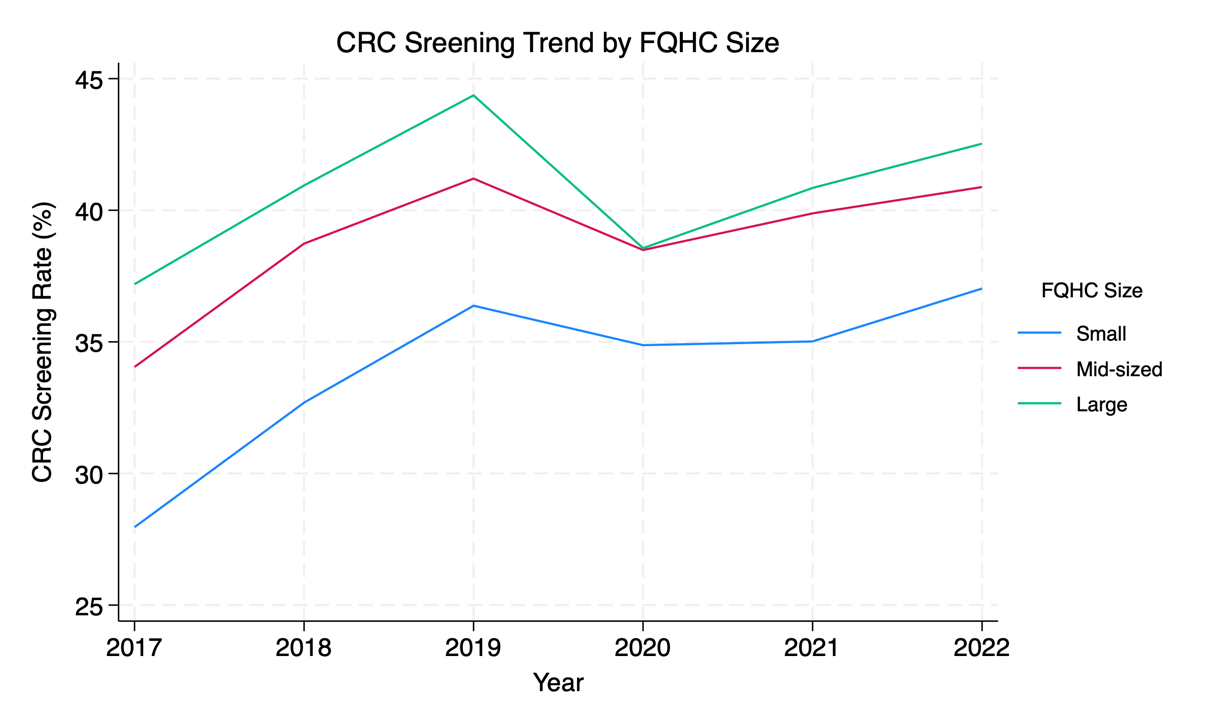
**

Note: Line graphs showing trends in CRC screening rates from 2017 to 2022, stratified by US region, FQHC size.

| **Table B1. Mixed Effects Model: Fully Adjusted with Main and Interaction Effects** | | | | | | | |
| --- | --- | --- | --- | --- | --- | --- | --- |
| Variable | Coef. | Std. err. | Wald’s z | p-value | [95% conf. interval] | | Sig |
| Time piece1 | 2.535 | 1.546 | 1.64 | .101 | -.496 | 5.566 |  |
| Time piece2 | 1.359 | 2.751 | 0.49 | .621 | -4.034 | 6.751 |  |
| Time piece3 | 1.196 | 1.528 | 0.78 | .434 | -1.798 | 4.19 |  |
| PCMH years | - | - | - | - | - | - |  |
| At least 1 | **5.702** | 1.752 | 3.25 | .001 | 2.268 | 9.136 | *** |
| All | **8.502** | 1.564 | 5.44 | 0 | 5.437 | 11.567 | *** |
| PCMHYears#  piece1 | - | - | - | - | - | - |  |
| At least 1 | -.106 | .715 | -0.15 | .882 | -1.508 | 1.295 |  |
| All | -.104 | .648 | -0.16 | .872 | -1.374 | 1.165 |  |
| PCMHYears#  piece2 | - | - | - | - | - | - |  |
| At least 1 | -.199 | 1.208 | -0.16 | .869 | -2.566 | 2.168 |  |
| All | -.386 | 1.094 | -0.35 | .724 | -2.531 | 1.758 |  |
| PCMHYears#  piece3 | - | - | - | - | - | - |  |
| At least 1 | **-1.624** | .618 | -2.63 | .009 | -2.834 | -.414 | *** |
| All | **-1.429** | .558 | -2.56 | .01 | -2.522 | -.336 | ** |
| Medicare | **.272** | .082 | 3.33 | .001 | .112 | .432 | *** |
| Medicare#  piece1 | -.009 | .04 | -0.24 | .812 | -.087 | .068 |  |
| Medicare#  piece2 | **.206** | .066 | 3.15 | .002 | .078 | .335 | *** |
| Medicare#  piece3 | **-.058** | .033 | -1.76 | .079 | -.124 | .007 | * |
| ContHTN | **.354** | .021 | 17.23 | 0 | .314 | .394 | *** |
| ContHTN#piece1 | .01 | .014 | 0.66 | .509 | -.019 | .038 |  |
| ContHTN#piece2 | -.016 | .029 | -0.55 | .581 | -.073 | .041 |  |
| ContHTN#piece3 | **.029** | .017 | 1.73 | .083 | -.004 | .063 | * |
| UncontDM | .02 | .025 | 0.82 | .413 | -.028 | .069 |  |
| UncontDM#piece1 | **-.04** | .017 | -2.34 | .019 | -.074 | -.006 | ** |
| UncontDM#piece2 | .008 | .032 | 0.24 | .809 | -.055 | .07 |  |
| UncontDM#piece3 | **-.06** | .018 | -3.27 | .001 | -.096 | -.024 | *** |
| Black Population | - | - | - | - | - | - |  |
| 10-20 | **-3.244** | 1.263 | -2.57 | .01 | -5.72 | -.768 | ** |
| >20 | **-2.979** | 1.161 | -2.57 | .01 | -5.254 | -.704 | ** |
| **Table B1. Mixed Effects Model: Fully Adjusted with Main and Interaction Effects** | | | | | | | |
| CRC Screening | Coef. | Std. err. | Wald’s z | p-value | [95% conf. interval] | | Sig |
| Blackpop#piece1 | - | - | - | - | - | - |  |
| 10-20 | .342 | .654 | 0.52 | .601 | -.939 | 1.624 |  |
| >20 | .462 | .53 | 0.87 | .384 | -.577 | 1.501 |  |
| Blackpop#piece2 | - | - | - | - | - | - |  |
| 10-20 | 1.545 | 1.1 | 1.40 | .16 | -.612 | 3.702 |  |
| >20 | .092 | .899 | 0.10 | .918 | -1.67 | 1.855 |  |
| Blackpop#piece3 | - | - | - | - | - | - |  |
| 10-20 | -.159 | .569 | -0.28 | .779 | -1.274 | .955 |  |
| >20 | -.04 | .46 | -0.09 | .931 | -.942 | .862 |  |
| Region of US | - | - | - | - | - | - |  |
| Midwest | **-5.396** | 1.623 | -3.32 | .001 | -8.578 | -2.215 | *** |
| South | **-5.951** | 1.508 | -3.95 | 0 | -8.907 | -2.996 | *** |
| West | **-4.697** | 1.592 | -2.95 | .003 | -7.817 | -1.576 | *** |
| region#piece1 | - | - | - | - | - | - |  |
| Midwest | .914 | .657 | 1.39 | .164 | -.374 | 2.202 |  |
| South | **1.368** | .624 | 2.19 | .028 | .145 | 2.591 | ** |
| West | -.107 | .659 | -0.16 | .871 | -1.398 | 1.183 |  |
| region#piece2 | - | - | - | - | - | - |  |
| Midwest | -.202 | 1.114 | -0.18 | .856 | -2.385 | 1.982 |  |
| South | -1.296 | 1.048 | -1.24 | .216 | -3.351 | .759 |  |
| West | .03 | 1.113 | 0.03 | .978 | -2.151 | 2.211 |  |
| region#piece3 | - | - | - | - | - | - |  |
| Midwest | -.287 | .571 | -0.50 | .616 | -1.405 | .832 |  |
| South | -.403 | .534 | -0.75 | .45 | -1.449 | .644 |  |
| West | .448 | .569 | 0.79 | .432 | -.668 | 1.564 |  |
| FQHC Size | - | - | - | - | - | - |  |
| Mid-sized | **1.66** | 1.045 | 1.59 | .112 | -.388 | 3.708 |  |
| Large | **2.675** | 1.308 | 2.05 | .041 | .112 | 5.238 | ** |
| size#piece1 | - | - | - | - | - | - |  |
| Mid-sized | -.834 | .523 | -1.60 | .111 | -1.858 | .19 |  |
| Large | -.904 | .63 | -1.44 | .151 | -2.139 | .33 |  |
| size#piece2 | - | - | - | - | - | - |  |
| Mid-sized | -.862 | .869 | -0.99 | .321 | -2.565 | .84 |  |
| Large | **-2.702** | 1.055 | -2.56 | .01 | -4.771 | -.633 | ** |
| size#piece3 | - | - | - | - | - | - |  |
| Mid-sized | .363 | .447 | 0.81 | .417 | -.513 | 1.239 |  |
| Large | **1.259** | .542 | 2.32 | .02 | .197 | 2.321 | ** |
| Medicaid | - | - | - | - | - | - |  |
| 25-75th | 1.004 | .955 | 1.05 | .293 | -.869 | 2.877 |  |
| **Table B1. Mixed Effects Model: Fully Adjusted with Main and Interaction Effects** | | | | | | | |
| CRC Screening | Coef. | Std. err. | Wald’s z | p-value | [95% conf. interval] | | Sig |
| >75th | -.183 | 1.222 | -0.15 | .881 | -2.578 | 2.211 |  |
| Medicaid#piece1 | - | - | - | - | - | - |  |
| 25-75th | -.195 | .528 | -0.37 | .711 | -1.231 | .84 |  |
| >75th | **1.214** | .669 | 1.82 | .069 | -.096 | 2.525 | * |
| Medicaid#piece2 | - | - | - | - | - | - |  |
| 25-75th | -.894 | .877 | -1.02 | .308 | -2.613 | .824 |  |
| >75th | -.916 | 1.14 | -0.80 | .422 | -3.151 | 1.319 |  |
| Medicaid#piece3 | - | - | - | - | - | - |  |
| 25-75th | .229 | .478 | 0.48 | .633 | -.709 | 1.166 |  |
| >75th | -.002 | .606 | -0.00 | .997 | -1.19 | 1.186 |  |
| Age Eligibility | -.107 | .072 | -1.49 | .136 | -.248 | .034 |  |
| AgeEl#piece1 | .029 | .034 | 0.84 | .4 | -.038 | .096 |  |
| AgeEl#piece2 | -.059 | .058 | -1.01 | .313 | -.173 | .055 |  |
| AgeEl#piece3 | .011 | .03 | 0.38 | .703 | -.047 | .07 |  |
| Constant | 9.591 | 3.025 | 3.17 | .002 | 3.661 | 15.52 | *** |

| Random-effects parameters | Estimate | Std. err. | [95 =% conf. interval] | |
| --- | --- | --- | --- | --- |
| sd(piece1) | 4.708 | .224 | 4.29 | 5.168 |
| sd(piece2) | 7.073 | .436 | 6.268 | 7.98 |
| sd(piece3) | 3.129 | .265 | 2.65 | 3.694 |
| sd(constant) | 16.067 | .39 | 15.321 | 16.85 |
| corr(piece1,piece2) | .148 | .084 | -.02 | .307 |
| corr(piece1,piece3) | -.21 | .082 | -.365 | -.045 |
| corr(piece1,constant) | -.479 | .036 | -.546 | -.407 |
| corr(piece2,piece3) | .086 | .11 | -.131 | .294 |
| corr(piece2,constant) | -.393 | .053 | -.492 | -.284 |
| corr(piece3,constant) | -.34 | .062 | -.455 | -.214 |
| sd(Residual) | 7.367 | .104 | 7.166 | 7.575 |

Note: *** p<.01, ** p<.05, * p<.1. Estimates derived from mixed-effects linear models with random intercepts at the FQHC level. Models incorporated piecewise time variables to assess changes in predicted CRC screening rates across distinct time phases and included interaction terms to examine whether predictor effects varied by time segment. Time segments are piece 1 (2017-2019), piece 2 (2019-2020), and piece 3 (2021-2022). All models were adjusted with appropriate variables listed in table. PCMH= Patient-Centered Medical Home; PCMH Years= Number of years a FQHC was reported to have PCMH recognition. ContHTN= Controlled hypertension; UncontDM= Uncontrolled diabetes; Blackpop= percentage of Black patients in population; AgeEL= percentage of age eligible adults; sd= standard deviation. The constant represents the predicted CRC screening rate in 2017 for FQHCs with baseline values on all covariates—i.e., reference categories for categorical predictors and zero for continuous variables.

| **Table B2. Adjusted Margins: CRC Screening Rates by PCMH Recognition Across Representative Thresholds of Organizational-Level Variables** | | | | |
| --- | --- | --- | --- | --- |
| **Variable** | **Level** | **PCMH Duration** | **Predicted Screening (%)** | **Margin Difference (vs. None)** |
| **Uncontrolled Diabetes** | Highest | None | 24.05 | - |
|  |  | 1 year | 31.78 | +7.73 |
|  |  | All years | 36.12 | +12.07 |
| **Controlled Hypertension** | Lowest | None | 27.52 | - |
|  |  | 1 year | 32.99 | +5.47 |
|  |  | All years | 36.37 | +8.85 |
| **Region** | South | None | 26.84 | - |
|  |  | 1 year | 32.51 | +5.67 |
|  |  | All years | 35.13 | +8.29 |
|  | Northeast | None | 30.11 | - |
|  |  | 1 year | 34.73 | +4.62 |
|  |  | All years | 39.02 | +8.91 |
| **FQHC Size** | Small | None | 25.96 | - |
|  |  | 1 year | 30.67 | +4.71 |
|  |  | All years | 33.41 | +7.45 |
|  | Large | None | 29.89 | - |
|  |  | 1 year | 35.21 | +5.32 |
|  |  | All years | 39.20 | +9.31 |
| **Black Population** | Lowest | None | 27.18 | - |
|  |  | 1 year | 31.92 | +4.74 |
|  |  | All years | 34.97 | +7.79 |
|  | Highest | None | 24.89 | - |
|  |  | 1 year | 30.84 | +5.95 |
|  |  | All years | 35.61 | +10.72 |

Note: Thresholds were selected to highlight adjusted margins at representative high and low values of key organizational-level variables, including chronic disease burden, regional location, racial composition, and size. “Margin Difference” reflects the difference in predicted CRC screening rates compared to FQHCs without PCMH recognition. All models included random intercepts for FQHC and were adjusted for percentage of age eligible adults and insurance mix. Full margins output available upon request.
